# 1-(1-Benzo­furan-2-yl)ethanone *O*-(2,6-di­fluoro­benz­yl)oxime

**DOI:** 10.1107/S1600536813034090

**Published:** 2013-12-24

**Authors:** Tomasz Kosmalski, Andrzej K. Gzella

**Affiliations:** a Department of Organic Chemistry, Ludwik Rydygier Collegium Medicum in Bydgoszcz, Nicolaus Copernicus University in Torun, ul. A. Jurasza 2, 85-089 Bydgoszcz, Poland; b Department of Organic Chemistry, Poznan University of Medical Sciences, ul. Grunwaldzka 6, 60-780 Poznań, Poland

## Abstract

In the title compound, C_17_H_13_F_2_NO_2_, the 2,2-di­fluoro­benz­yloxy residue assumes an *E* configuration with respect to the benzo­furan system. The benzene ring makes a dihedral angle of 61.70 (4)° with the fused ring system (r.m.s. deviation = 0.008 Å). In the crystal, mol­ecules are connected by weak C—H⋯F hydrogen bonds into chains extending parallel to the *b-*axis direction.

## Related literature   

For background to anti­fungal agents, see: Benedetti & Bani (1999 ▶); Sheehan *et al.* (1999 ▶). For the biological activity of oximes and their ethers, see: Attia *et al.* (2013 ▶); De Luca (2006 ▶); Emami *et al.* (2004 ▶); Karakurt *et al.* (2001 ▶); Massolini *et al.* (1993 ▶); Mixich & Thiele (1985 ▶). For the synthesis of the title compound, see: Demirayak *et al.* (2002 ▶).
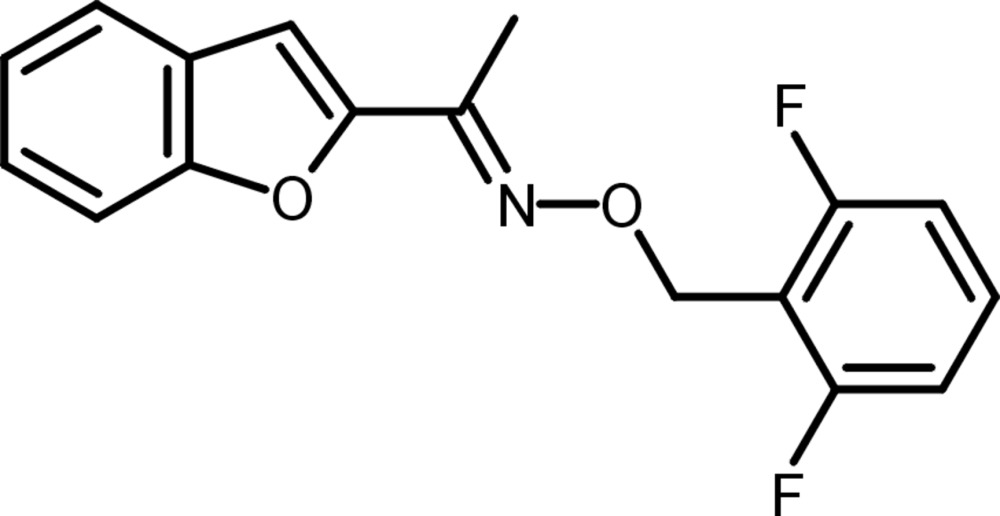


## Experimental   

### 

#### Crystal data  

C_17_H_13_F_2_NO_2_
*M* *_r_* = 301.28
Monoclinic, 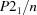

*a* = 7.36652 (17) Å
*b* = 17.0314 (4) Å
*c* = 11.2047 (2) Å
β = 90.020 (2)°
*V* = 1405.76 (5) Å^3^
*Z* = 4
Mo *K*α radiation
μ = 0.11 mm^−1^
*T* = 130 K
0.35 × 0.15 × 0.12 mm

#### Data collection  

Agilent Xcalibur Atlas diffractometer
Absorption correction: multi-scan (*CrysAlis PRO*; Agilent, 2011 ▶) *T*_min_ = 0.992, *T*_max_ = 1.000
24335 measured reflections
3548 independent reflections
2887 reflections with *I* > 2σ(*I*)
*R*_int_ = 0.033

#### Refinement  

*R*[*F* ^2^ > 2σ(*F* ^2^)] = 0.041
*wR*(*F* ^2^) = 0.099
*S* = 1.03
3548 reflections
200 parameters
H-atom parameters constrained
Δρ_max_ = 0.26 e Å^−3^
Δρ_min_ = −0.24 e Å^−3^

### 

Data collection: *CrysAlis PRO* (Agilent, 2011 ▶); cell refinement: *CrysAlis PRO*; data reduction: *CrysAlis PRO*; program(s) used to solve structure: *SHELXS97* (Sheldrick, 2008 ▶); program(s) used to refine structure: *SHELXL97* (Sheldrick, 2008 ▶); molecular graphics: *ORTEP-3 for Windows* (Farrugia, 2012 ▶); software used to prepare material for publication: *WinGX* (Farrugia, 2012 ▶) and *PLATON* (Spek, 2009 ▶).

## Supplementary Material

Crystal structure: contains datablock(s) I, publication_text. DOI: 10.1107/S1600536813034090/zs2281sup1.cif

Structure factors: contains datablock(s) I. DOI: 10.1107/S1600536813034090/zs2281Isup2.hkl

Supporting information file. DOI: 10.1107/S1600536813034090/zs2281Isup3.cml

Additional supporting information:  crystallographic information; 3D view; checkCIF report

## Figures and Tables

**Table 1:** Hydrogen-bond geometry (Å, °)

*D*—H⋯*A*	*D*—H	H⋯*A*	*D*⋯*A*	*D*—H⋯*A*
C7—H7⋯F22^i^	0.93	2.54	3.3537 (16)	147

Symmetry code: (i) 
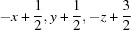
.

**Fig. 1.**
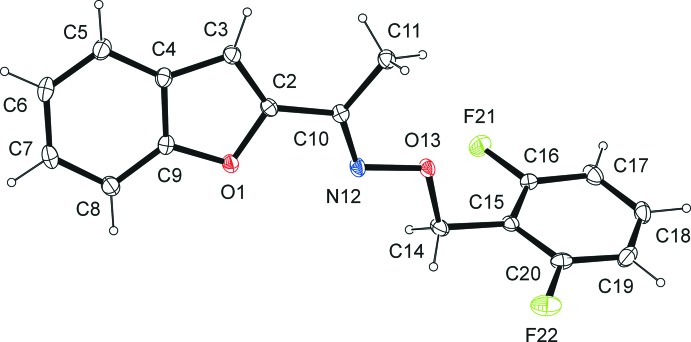
The molecular structure of the title compound showing the atom labelling scheme. Non-H atoms are drawn as 30% probability displacement ellipsoids and H atoms are shown as small spheres of arbitrary radius.

**Fig. 2.**
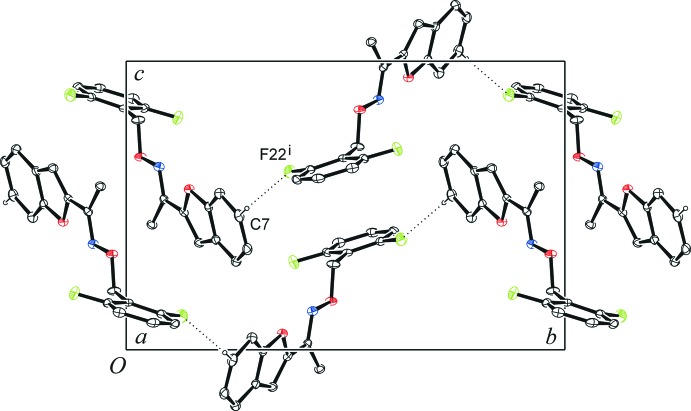
The hydrogen bonding (dotted lines) in the title structure. For symmetry code (i), see Table 1. H atoms not involved in hydrogen-bonding have been omitted for clarity.

## supplementary crystallographic information

### 1. Comment

The increase in fungal infections and the gained resistance to the currently used drugs in recent years directed the studies on obtaining new antifungal drugs (Benedetti & Bani, 1999). After the discovery of oxiconazole (Sheehan *et al.*, 1999), ether oximes became of interest and a number of oximes were synthesized and found to be active against fungi (Attia *et al.*, 2013; De Luca, 2006; Emami *et al.*, 2004; Karakurt *et al.*, 2001; Massolini *et al.*, 1993; Mixich & Thiele, 1985). The crystal structure investigation of the title compound was undertaken to confirm the *E* configuration of the molecule, proposed on the basis of spectroscopic data.

The molecular structure of the title compound and the atom-labelling scheme is illustrated in Fig. 1. In this compound, the nine-membered benzofuran system is planar with an r.m.s. deviation of 0.0083 Å. The 2,6-difluorobenzyloxy moiety is in the *E* configuration with respect to the benzofuran system [torsion angle C2—C10—N12—O13: 178.89 (9)°]. The C10—N12 bond is antiperiplanar in relation to the O13—C14 bond [torsion angle C10—N12—O13—C14: 176.13 (10)°]. A similar observation has been made for bonds N12—O13 and C14—C15 [torsion angle N12—O13—C14—C15: 170.08 (10)°]. The planar benzofuran system and the phenyl ring form a dihedral angle of 61.70 (4)°.

The molecular packing in the crystal lattice is stabilized by possible C7—H7···F22^i^ non-classical intermolecular hydrogen bonds (Table 1) which link molecules into chains lying parallel to the *b* axis (Fig. 2).

### 2. Experimental

1-(1-Benzofuran-2-yl)ethanone O-(2,6-difluorobenzyl)oxime was synthesized from 1-(benzofuran-2-yl)ethanone oxime and 2,6-difluorobenzyl bromide, according to the literature procedure of Demirayak *et al.* (2002). Crystals were obtained after crystallization from ethanol.

### 3. Refinement

All H atoms were placed in idealized positions and were refined within the riding model approximation: C_methyl_—H = 0.96 Å, C_methylene_—H = 0.97 Å, C(*sp*^2^)—H = 0.93 Å; *U*_iso_(H) = 1.2*U*_eq_(C) or 1.5*U*_eq_(C) for methyl H. The methyl group was refined as a rigid group which was allowed to rotate.

### Crystal data

**Table:**

C_17_H_13_F_2_NO_2_	*F*(000) = 624
*M**_r_* = 301.28	*D*_x_ = 1.424 Mg m^−^^3^
Monoclinic, *P*2_1_/*n*	Melting point = 346–348 K
Hall symbol: -P 2yn	Mo *K*α radiation, λ = 0.71073 Å
*a* = 7.36652 (17) Å	Cell parameters from 10469 reflections
*b* = 17.0314 (4) Å	θ = 2.2–29.1°
*c* = 11.2047 (2) Å	µ = 0.11 mm^−^^1^
β = 90.020 (2)°	*T* = 130 K
*V* = 1405.76 (5) Å^3^	Indefinite, colourless
*Z* = 4	0.35 × 0.15 × 0.12 mm

### Data collection

**Table:**

Agilent Xcalibur Atlas diffractometer	3548 independent reflections
Radiation source: Enhance (Mo) X-ray Source	2887 reflections with *I* > 2σ(*I*)
Graphite monochromator	*R*_int_ = 0.033
Detector resolution: 10.3088 pixels mm^-1^	θ_max_ = 29.1°, θ_min_ = 2.2°
ω scans	*h* = −9→10
Absorption correction: multi-scan (*CrysAlis PRO*; Agilent, 2011)	*k* = −23→22
*T*_min_ = 0.992, *T*_max_ = 1.000	*l* = −14→15
24335 measured reflections	

### Refinement

**Table:**

Refinement on *F*^2^	Primary atom site location: structure-invariant direct methods
Least-squares matrix: full	Secondary atom site location: difference Fourier map
*R*[*F*^2^ > 2σ(*F*^2^)] = 0.041	Hydrogen site location: inferred from neighbouring sites
*wR*(*F*^2^) = 0.099	H-atom parameters constrained
*S* = 1.03	*w* = 1/[σ^2^(*F*_o_^2^) + (0.0434*P*)^2^ + 0.5216*P*] where *P* = (*F*_o_^2^ + 2*F*_c_^2^)/3
3548 reflections	(Δ/σ)_max_ < 0.001
200 parameters	Δρ_max_ = 0.26 e Å^−^^3^
0 restraints	Δρ_min_ = −0.24 e Å^−^^3^

### Special details

**Table:**

Geometry. All s.u.'s (except the s.u. in the dihedral angle between two l.s. planes) are estimated using the full covariance matrix. The cell s.u.'s are taken into account individually in the estimation of s.u.'s in distances, angles and torsion angles; correlations between s.u.'s in cell parameters are only used when they are defined by crystal symmetry. An approximate (isotropic) treatment of cell s.u.'s is used for estimating s.u.'s involving l.s. planes.
Refinement. Refinement of *F*^2^ against ALL reflections. The weighted *R*-factor *wR* and goodness of fit *S* are based on *F*^2^, conventional *R*-factors *R* are based on *F*, with *F* set to zero for negative *F*^2^. The threshold expression of *F*^2^ > 2σ(*F*^2^) is used only for calculating *R*-factors(gt) *etc*. and is not relevant to the choice of reflections for refinement. *R*-factors based on *F*^2^ are statistically about twice as large as those based on *F*, and *R*- factors based on ALL data will be even larger.

### Fractional atomic coordinates and isotropic or equivalent isotropic displacement parameters (Å^2^)

**Table:**

	*x*	*y*	*z*	*U*_iso_*/*U*_eq_	
O1	0.16108 (11)	0.14365 (5)	0.55614 (7)	0.0225 (2)	
C2	0.01949 (16)	0.13157 (7)	0.47691 (11)	0.0202 (2)	
C3	0.05125 (18)	0.16689 (7)	0.37076 (11)	0.0246 (3)	
H3	−0.0245	0.1667	0.3044	0.030*	
C4	0.22538 (17)	0.20490 (7)	0.38062 (11)	0.0228 (3)	
C5	0.3345 (2)	0.25081 (8)	0.30607 (12)	0.0292 (3)	
H5	0.2964	0.2639	0.2295	0.035*	
C6	0.50053 (19)	0.27631 (8)	0.34892 (13)	0.0302 (3)	
H6	0.5750	0.3065	0.3001	0.036*	
C7	0.55844 (18)	0.25753 (8)	0.46421 (13)	0.0294 (3)	
H7	0.6711	0.2753	0.4902	0.035*	
C8	0.45207 (18)	0.21312 (8)	0.54084 (13)	0.0268 (3)	
H8	0.4893	0.2009	0.6179	0.032*	
C9	0.28652 (16)	0.18797 (7)	0.49543 (11)	0.0208 (3)	
C10	−0.13574 (16)	0.08676 (7)	0.52060 (11)	0.0199 (2)	
C11	−0.27542 (17)	0.05873 (8)	0.43335 (11)	0.0246 (3)	
H11A	−0.2468	0.0062	0.4088	0.037*	
H11B	−0.2761	0.0926	0.3649	0.037*	
H11C	−0.3929	0.0594	0.4704	0.037*	
N12	−0.13799 (14)	0.07490 (6)	0.63388 (9)	0.0226 (2)	
O13	−0.29434 (11)	0.03251 (5)	0.66817 (8)	0.0240 (2)	
C14	−0.28904 (17)	0.02743 (9)	0.79613 (11)	0.0275 (3)	
H14A	−0.2683	0.0789	0.8305	0.033*	
H14B	−0.1917	−0.0072	0.8213	0.033*	
C15	−0.46849 (16)	−0.00434 (7)	0.83627 (10)	0.0200 (2)	
C16	−0.62337 (17)	0.04170 (7)	0.83715 (11)	0.0222 (3)	
C17	−0.79223 (18)	0.01540 (9)	0.87023 (12)	0.0287 (3)	
H17	−0.8924	0.0486	0.8687	0.034*	
C18	−0.80877 (19)	−0.06188 (9)	0.90588 (12)	0.0316 (3)	
H18	−0.9222	−0.0813	0.9272	0.038*	
C19	−0.6595 (2)	−0.11065 (8)	0.91027 (12)	0.0332 (3)	
H19	−0.6704	−0.1624	0.9358	0.040*	
C20	−0.49296 (19)	−0.08056 (7)	0.87563 (11)	0.0252 (3)	
F21	−0.60530 (12)	0.11776 (5)	0.80385 (8)	0.0363 (2)	
F22	−0.34453 (13)	−0.12699 (5)	0.88276 (8)	0.0428 (2)	

### Atomic displacement parameters (Å^2^)

**Table:**

	*U*^11^	*U*^22^	*U*^33^	*U*^12^	*U*^13^	*U*^23^
O1	0.0189 (4)	0.0270 (5)	0.0216 (4)	−0.0037 (3)	0.0001 (3)	0.0006 (3)
C2	0.0188 (6)	0.0209 (6)	0.0209 (6)	0.0000 (4)	−0.0008 (5)	−0.0047 (4)
C3	0.0256 (6)	0.0270 (6)	0.0211 (6)	−0.0050 (5)	−0.0012 (5)	−0.0009 (5)
C4	0.0255 (6)	0.0196 (6)	0.0234 (6)	−0.0031 (5)	0.0016 (5)	−0.0029 (4)
C5	0.0362 (8)	0.0257 (6)	0.0257 (7)	−0.0078 (6)	0.0016 (6)	0.0011 (5)
C6	0.0326 (7)	0.0229 (6)	0.0350 (7)	−0.0088 (5)	0.0080 (6)	−0.0012 (5)
C7	0.0219 (6)	0.0255 (6)	0.0407 (8)	−0.0061 (5)	0.0002 (6)	−0.0051 (5)
C8	0.0231 (6)	0.0277 (7)	0.0295 (7)	−0.0016 (5)	−0.0023 (5)	−0.0006 (5)
C9	0.0207 (6)	0.0174 (5)	0.0244 (6)	−0.0004 (4)	0.0041 (5)	−0.0018 (4)
C10	0.0188 (6)	0.0203 (6)	0.0206 (6)	0.0013 (4)	0.0020 (5)	−0.0028 (4)
C11	0.0253 (6)	0.0271 (6)	0.0213 (6)	−0.0052 (5)	−0.0005 (5)	−0.0023 (5)
N12	0.0160 (5)	0.0292 (6)	0.0225 (5)	−0.0043 (4)	0.0033 (4)	−0.0012 (4)
O13	0.0174 (4)	0.0369 (5)	0.0178 (4)	−0.0076 (4)	0.0023 (3)	0.0005 (4)
C14	0.0184 (6)	0.0468 (8)	0.0173 (6)	−0.0052 (5)	−0.0012 (5)	0.0029 (5)
C15	0.0181 (6)	0.0286 (6)	0.0134 (5)	−0.0020 (5)	−0.0008 (4)	0.0004 (4)
C16	0.0231 (6)	0.0243 (6)	0.0194 (6)	−0.0022 (5)	−0.0023 (5)	0.0030 (4)
C17	0.0185 (6)	0.0426 (8)	0.0249 (7)	−0.0001 (5)	0.0006 (5)	−0.0031 (6)
C18	0.0271 (7)	0.0467 (8)	0.0209 (6)	−0.0161 (6)	0.0027 (5)	−0.0005 (6)
C19	0.0520 (9)	0.0264 (7)	0.0212 (6)	−0.0149 (6)	−0.0030 (6)	0.0039 (5)
C20	0.0317 (7)	0.0261 (6)	0.0179 (6)	0.0044 (5)	−0.0037 (5)	−0.0015 (5)
F21	0.0375 (5)	0.0264 (4)	0.0449 (5)	0.0016 (3)	−0.0033 (4)	0.0099 (3)
F22	0.0504 (6)	0.0360 (5)	0.0421 (5)	0.0204 (4)	−0.0081 (4)	−0.0012 (4)

### Geometric parameters (Å, º)

**Table:**

O1—C9	1.3736 (14)	C11—H11B	0.9600
O1—C2	1.3848 (15)	C11—H11C	0.9600
C2—C3	1.3534 (17)	N12—O13	1.4127 (13)
C2—C10	1.4594 (17)	O13—C14	1.4369 (15)
C3—C4	1.4410 (18)	C14—C15	1.4977 (17)
C3—H3	0.9300	C14—H14A	0.9700
C4—C9	1.3929 (18)	C14—H14B	0.9700
C4—C5	1.3986 (18)	C15—C20	1.3828 (18)
C5—C6	1.384 (2)	C15—C16	1.3844 (17)
C5—H5	0.9300	C16—F21	1.3547 (14)
C6—C7	1.397 (2)	C16—C17	1.3732 (18)
C6—H6	0.9300	C17—C18	1.381 (2)
C7—C8	1.3870 (19)	C17—H17	0.9300
C7—H7	0.9300	C18—C19	1.379 (2)
C8—C9	1.3890 (18)	C18—H18	0.9300
C8—H8	0.9300	C19—C20	1.385 (2)
C10—N12	1.2853 (16)	C19—H19	0.9300
C10—C11	1.4972 (17)	C20—F22	1.3517 (15)
C11—H11A	0.9600		
			
C9—O1—C2	105.72 (9)	H11A—C11—H11B	109.5
C3—C2—O1	111.52 (11)	C10—C11—H11C	109.5
C3—C2—C10	131.52 (11)	H11A—C11—H11C	109.5
O1—C2—C10	116.92 (10)	H11B—C11—H11C	109.5
C2—C3—C4	106.64 (11)	C10—N12—O13	111.08 (10)
C2—C3—H3	126.7	N12—O13—C14	106.28 (9)
C4—C3—H3	126.7	O13—C14—C15	107.32 (10)
C9—C4—C5	118.79 (12)	O13—C14—H14A	110.3
C9—C4—C3	105.39 (11)	C15—C14—H14A	110.3
C5—C4—C3	135.82 (13)	O13—C14—H14B	110.3
C6—C5—C4	118.45 (13)	C15—C14—H14B	110.3
C6—C5—H5	120.8	H14A—C14—H14B	108.5
C4—C5—H5	120.8	C20—C15—C16	114.96 (11)
C5—C6—C7	121.23 (12)	C20—C15—C14	123.38 (12)
C5—C6—H6	119.4	C16—C15—C14	121.67 (11)
C7—C6—H6	119.4	F21—C16—C17	118.38 (12)
C8—C7—C6	121.65 (12)	F21—C16—C15	117.30 (11)
C8—C7—H7	119.2	C17—C16—C15	124.32 (12)
C6—C7—H7	119.2	C16—C17—C18	117.97 (13)
C7—C8—C9	115.95 (13)	C16—C17—H17	121.0
C7—C8—H8	122.0	C18—C17—H17	121.0
C9—C8—H8	122.0	C19—C18—C17	120.94 (12)
O1—C9—C8	125.36 (12)	C19—C18—H18	119.5
O1—C9—C4	110.72 (10)	C17—C18—H18	119.5
C8—C9—C4	123.92 (12)	C18—C19—C20	118.26 (12)
N12—C10—C2	115.07 (11)	C18—C19—H19	120.9
N12—C10—C11	125.85 (11)	C20—C19—H19	120.9
C2—C10—C11	119.08 (11)	F22—C20—C15	117.55 (12)
C10—C11—H11A	109.5	F22—C20—C19	118.92 (12)
C10—C11—H11B	109.5	C15—C20—C19	123.51 (12)
			
C9—O1—C2—C3	−0.51 (13)	O1—C2—C10—C11	−168.36 (10)
C9—O1—C2—C10	−178.77 (10)	C2—C10—N12—O13	178.89 (9)
O1—C2—C3—C4	−0.06 (14)	C11—C10—N12—O13	−1.33 (17)
C10—C2—C3—C4	177.86 (12)	C10—N12—O13—C14	−176.13 (10)
C2—C3—C4—C9	0.60 (14)	N12—O13—C14—C15	170.08 (10)
C2—C3—C4—C5	−179.29 (15)	O13—C14—C15—C20	105.09 (13)
C9—C4—C5—C6	1.18 (19)	O13—C14—C15—C16	−75.36 (15)
C3—C4—C5—C6	−178.95 (14)	C20—C15—C16—F21	177.47 (11)
C4—C5—C6—C7	−0.5 (2)	C14—C15—C16—F21	−2.12 (17)
C5—C6—C7—C8	−0.4 (2)	C20—C15—C16—C17	−2.02 (18)
C6—C7—C8—C9	0.70 (19)	C14—C15—C16—C17	178.39 (12)
C2—O1—C9—C8	−179.21 (12)	F21—C16—C17—C18	−178.97 (11)
C2—O1—C9—C4	0.91 (13)	C15—C16—C17—C18	0.5 (2)
C7—C8—C9—O1	−179.90 (11)	C16—C17—C18—C19	1.2 (2)
C7—C8—C9—C4	−0.03 (19)	C17—C18—C19—C20	−1.3 (2)
C5—C4—C9—O1	178.96 (11)	C16—C15—C20—F22	−176.55 (11)
C3—C4—C9—O1	−0.95 (13)	C14—C15—C20—F22	3.03 (18)
C5—C4—C9—C8	−0.92 (19)	C16—C15—C20—C19	1.93 (18)
C3—C4—C9—C8	179.17 (12)	C14—C15—C20—C19	−178.49 (12)
C3—C2—C10—N12	−166.39 (13)	C18—C19—C20—F22	178.09 (12)
O1—C2—C10—N12	11.44 (16)	C18—C19—C20—C15	−0.4 (2)
C3—C2—C10—C11	13.8 (2)		

### Hydrogen-bond geometry (Å, º)

**Table:**

*D*—H···*A*	*D*—H	H···*A*	*D*···*A*	*D*—H···*A*
C7—H7···F22^i^	0.93	2.54	3.3537 (16)	147

Symmetry code: (i) −*x*+1/2, *y*+1/2, −*z*+3/2.
